# Deep convolutional neural network for automatic discrimination between *Fragaria* × *Ananassa* flowers and other similar white wild flowers in fields

**DOI:** 10.1186/s13007-018-0332-5

**Published:** 2018-07-27

**Authors:** Ping Lin, Du Li, Zhiyong Zou, Yongming Chen, Shanchao Jiang

**Affiliations:** 1 College of Electrical Engineering, Yancheng Institute of Technology, No.1 Middle Road Hope Avenue, Yancheng, 224051 Jiangsu Province People’s Republic of China; 2 College of Mechanical and Electrical Engineering, Sichuan Agricultural University, Ya’an, 625014 People’s Republic of China

**Keywords:** *Fragaria* × *ananassa*, Flower, Identify, Convolutional neural network

## Abstract

**Background:**

The images of different flower species had small inter-class variations across different classes as well as large intra-class variations within a class. Flower classification techniques are mainly based on the features of color, shape and texture, however, the procedure always involves too many heuristics as well as manual labor to tweak parameters, which often leads to datasets with poor qualitative and quantitative measures. The current study proposed a deep architecture of convolutional neural network (CNN) for the purposes of improving the accuracy of identifying the white flowers of *Fragaria* × *ananassa* from other three wild flower species of *Androsace umbellata* (Lour.) Merr., *Bidens pilosa* L. and *Trifolium repens* L. in fields.

**Results:**

The explored CNN architecture consisted of eightfolds of learnable weights including 5 convolutional layers and 3 fully connected layers, which received a true color 227 × 227 pixels flower image as its input. The developed CNN detector was able to classify the instances of flowers at overall average accuracies of 99.2 and 95.0% in the training and test procedure, respectively. The state-of-the-art CNN model was compared with the classical models of the scale-invariant feature transform (SIFT) features and the pyramid histogram of orientated gradient (PHOG) features combined with the multi-class support vector machine (SVM) algorithm. The proposed model turned out to be much more accurate than the traditional models of SIFT + SVM at overall average accuracies of 82.9 and 55.6% in the training and test procedure and PHOG + SVM at overall average accuracies of 78.3 and 63.1%, respectively.

**Conclusions:**

The proposed state-of-the-art CNN method demonstrates that artificial intelligence is capable of precise classification of the white flower images, whose accuracy is comparable to traditional algorithms. The presented algorithm can be further used for the discrimination of white wild flowers in fields.

## Background

The distribution and yield of flowers in fields are of significant agronomic importance, being the precursor of quality of fruits and seeds [8, 24, 33]. Despite exploiting several systems to manage them in the past decade, the development of fine flower detection systems is still one of the important issues in modern smart agriculture [13, 15, 30]. Discrimination of flower species is a difficult mission for the current detection algorithms, because there are great variations in viewpoint and scale, illumination, partial occlusions, multiple instances etc. in the typical flower images [6, 22, 34]. The complex backgrounds also make the discrimination task more difficult, for risking probably discriminating background scenes rather than the object itself [18, 23]. Perhaps the greatest challenge originates from the intra-category versus inter-category diversification, i.e. there is a slighter difference between images of different categories than within a category itself, and yet subtle variation between instances determine their species [5, 6, 19].

The traditional flower classification is mainly based on the three features: color, shape and texture. This case requires people to select features for classification. An approach using the various features including color, shape, and texture was proposed to distinguish the flower categories [25]. However, Nilsback’s approach only used a single scale to extract the flower features. The multiple scale features such as scale-invariant feature transform (SIFT) and Gabor-based descriptors were proposed to improve the identification accuracy. A new method using multiple color SIFT features was proposed to improve the performance of flower image classification [32]. Guru et al. [14] presented a model extracting the grey-grade co-occurrence matrix, color texture moments and Gabor descriptors from the flower images for dealing with the flower classification issues [14]. In order to fuse multiple features from one image, the visual vocabulary method is presented to map certain feature through the clustering process and the image can be represented by histogram representation based on independent features. Hu et al. [16] explored a visual vocabulary methods to describe the four kinds of color-SIFT features for the discrimination of flower images [16]. In addition to improving recognition accuracy in feature extraction algorithms, scholars also attempted to improve recognition performance on feature recognition algorithms. A marginalized kernel algorithm was developed by utilizing the responses of the logistic regression-based fusion model for detecting the flower images [11]. Those models have demonstrated effectiveness for image classification to a certain degree. However, plenty of parameters of feature extraction algorithms needed to be tuned and many different types of features needed to be reshaped to species semantics. The spatial information and correlations sometimes were neglected when considering the local features. Besides, the encoding of local features causes some information loss which also hinders the final image classification performance. These algorithms always involve too many heuristics as well as manual labor to tweak parameters according to the domain to reach a decent level of accuracy.

Recently, the biologically inspired two-dimensional convolutional neural network (CNN), has been used as an effective tool for extracting the image features, giving superior accuracy on the classification, segmentation and retrieval tasks [21]. The basic idea of CNN is to build invariance properties into neural networks by creating models that are invariant to certain inputs transformation [35]. The proposed CNN architecture consists of alternatively stacked convolutional layers and spatial pooling layers. The convolutional layer is used to extract feature maps by linear convolutional filters followed by nonlinear activation functions such as the rectified linear units. Spatial pooling is performed to group the local features together from spatially adjacent pixels, which is typically done to improve the robustness to slight deformations of objects [10]. Our network consists of eightfolds of units which is similar to the AlexNet network structures [27] with learnable weights: 5 convolutional layers, and 3 fully connected layers. The convolutional layers and the max pooling layers in the whole CNN are presented to cope with the deep-level information of flower images. The intractable over-fitting case in the process of determining the characteristic parameters of network is solved by the stochastic gradient descent methods. The classical algorithms of SIFT and pyramid histogram of oriented gradients (PHOG) [4] combined with the multi-class support vector machine (SVM) [3] are compared with the state-of-the-art algorithm using multi-level convolutional architecture of CNN on the flower dataset to exhibit the advantage of the proposed architecture.

One of the main goals is that we want to build an artificial intelligent flower recognition system to accurately and automatically distinguish different species of flowers in the *Fragaria* × *Ananassa* fields. The presented system transferred the true color 227 × 227 pixels white flower images to 8 layers with learnable weights including 5 convolutional layers and 3 fully connected layers. Therefore, the input level has 51,529 neuron units at the beginning, and the following convolutional layers have a set of 96 filters. The subsampling layers contain rectified linear units layers and pooling layers. The final level is the fully connected layer with 4 neurons. The intractable over-fitting problem in determining the characteristic parameters of the network is solved by the stochastic gradient descent method. To this end, our team has set up a CNN architecture to recognize flower dataset which consists one flower species of *Fragaria* × *ananassa* and other three different wild flower species of *Androsace umbellata* (Lour.) Merr., *Bidens pilosa* L. and *Trifolium repens* L. There are blur, scale-variant, intra-class variant and inter-class similar objects among the experimental image dataset. The photographs of flowers are all captured in natural settings with rich and complex backgrounds. Although the background usually serves as distractor to detection model, sometimes it can supply useful information, so background content is also considered as the feature information for detection target. The rest of the paper is organized as follows: firstly, we presented the experimental data and device; The experimental methods are introduced subsequently; Then, the experimental results are analyzed and discussed. The conclusions are drawn finally.

## Experiment

### Experiment data

The experimental database composes of four distinct flower varieties of *Androsace umbellata* (Lour.) Merr., *Bidens pilosa* L., *Trifolium repens* L. and *Fragaria* × *ananassa.* These photos of white flowers were taken from the digital cameras in wild. The flower objects with petals and sepals were cropped individually from the raw digital photos by hand. There are blur, scale-variant, intra-class variant and inter-class similar objects among the experimental image dataset. The photographs of flowers were all captured in natural settings with rich and complex backgrounds. Although the background usually serves as distractor to detection model, sometimes it can supply useful information, so background content is also considered as the feature information for detection target. Some primary properties of these white flowers are summarized in Table 1. There are a total 400 flower images in the database, where each variety contains 100 images. For modeling the relationship between the flower features and the corresponding logical attributes, the experiment employed 60 and 40 images for both training and test aims for each type, respectively.

**Table 1:** Summary of four white flower species of *Androsace umbellata* (Lour.) Merr., *Bidens pilosa* L., *Trifolium repens* L. and *Fragaria* × *ananassa*

Family	Scientific name	Characteristics	Distribution and habitat	Image
Primulaceae	*Androsace umbellata* (Lour.) Merr.	Corolla regular (salverform or funnel-shaped), white, 4–6 mm broad; petals 5, quite round, touching each other, 4–6 mm in diameter, with a yellow eye. Calyx barely shorter than the corolla,3–4 mm diameter, 5-lobed, green; calyx broadly campanulate to subglobose or hemispheric, not keeled, glabrous, pilose, or puberulent; anthers subsessile. Inflorescence umbels [26]	Widely distributed in the mountains of central Asia, the Caucasus, and the southern and central European mountain systems, particularly the Alps and the Pyrenees. The species and their varieties bear vernacular names based on their characteristics. For example, they are known by such names as Rock Jasmine in English, Dian Dimei in China and Bom Maj I in Korean. They can be found in open grassy areas, roadsides, crops, pastures, gardens, shady places or near streams, ditches or moist embankments	
Asteraceae	*Bidens pilosa* L.	Corolla regular (radiate strap-shaped), white or cream, 5–15 mm broad; 5–8 ray petals, tubular bright yellow or orange disk florets in the center, 3.5–5 mm long. Inflorescence an isolated or grouped pedunculated capitula, emerging from the leaf axil [2]	It is thought to originate in South America and subsequently spread all over the world. The species and their varieties bear vernacular names based on their characteristics. For example, the species are known by such names as Spanish needles, Beggar’s ticks, Devil’s needles, Cobbler’s pegs, Broom stick and Pitchforks in English and some other languages because of their sticky achenes and are sometimes known as Xian Fengcao (“all bountiful grass”) in Chinese because of their prosperous growth. They can invade roadsides, crops, pastures, gardens, disturbed areas, fallow lands and urban open space	
Fabaceae	*Trifolium repens* L.	Corolla zygomorphic, white (sometimes slightly reddish), later brownish, 8–10 mm long, fused at base. Petals 5; the upstanding the ‘standard’, the lateral two the ‘wings’, the lower two united to form the ‘keel’, overall shape of corolla being butterfly-like. Calyx 5-lobed, glabrous. Stamens 10. A single carpel. Inflorescence a long-stalked, densely globose head, flowers fragrant [31]	Native to Europe and central Asia and has become one of the most widely distributed legumes in the world. The species are sometimes known as the White clover, Dutch clover and Ladino clover. The species thrive best in a cool, moist climate in soils with ample lime, phosphate, and potash. In general, it is best adapted to clay and silt soils in humid and irrigated areas. It grows successfully on sandy soils with a high-water table or irrigated droughty soils when adequately fertilized	
Rosaceae	*Fragaria* × *ananassa*	Corolla regular (actinomorphic), white, 12–18 mm broad; petals 5, quite round, touching each other or covering edges of each other, 4–6 mm long. Calyx 5-lobed; with epicalyx. Stamens 20. Gynoecium separate, pistils several. Receptacle glabrous. Inflorescence a lax cyme [1]	Firstly bred via a cross of *Fragaria virginiana* from eastern North America and *Fragaria chiloensis* from Chile. The species are sometimes known as the Garden strawberry, Fraisier des bois. The species is cultivated worldwide for its fruit. The habitat is along trails, roadsides, embankments, hillsides, stone- and gravel-laid paths and roads, meadows, young woodlands, sparse forest, woodland edges and clearings	

### Experimental devices

The classification algorithm of CNN was trained on the flower image dataset with a hardware solution of a Alienware 17 R4 laptop (DELL, USA) consisting of a NVIDIA GeForce GTX 1070 integrated RAMDAC 16 GB graphics card and Intel Core(TM) i7-6700H CPU. The algorithms were performed in Matlab R2017a (The Math Works, Natick, USA) on Windows 10 (Microsoft, USA) operating system. Caffe originally developed by the Berkeley vision and learning center was used as the deep learning framework [17]. The universal purpose computing on graphics processing units with NVIDIA GPUs using the parallel computing platform and application programming interface CUDA 8 with the deep neural network library CUDNN 7 were supported by Caffe. In our experiment, we took advantage of the NVIDIA GTX 1070 graphics card with 8 GB memory and 1024 kernels.

## Methods

### Scale invariant feature transform (SIFT) descriptor

The algorithm of SIFT intends to extract distinctive invariant features to represent the image. It uses the difference of Gaussian function of $DoG\left( {x,y,\sigma } \right)$ in the scale space to discover potential interest points:

1 $$DoG\left( {x,y,\sigma } \right) = \left( {G\left( {x,y,\xi \sigma } \right) - G\left( {x,y,\sigma } \right)} \right) \otimes I\left( {x,y} \right)$$

where, the symbol $\otimes$ denotes convolution operator, $\xi$ is a constant, $\sigma$ is the scale factor, $I\left( {x,y} \right)$ is the given input image and $G\left( {x,y,\sigma } \right) = \frac{1}{{2\pi \sigma^{2} }}e^{{ - \frac{{x^{2} + y^{2} }}{{\sigma^{2} }}}}$. The local extrema of $DoG\left( {x,y,\sigma } \right)$ are determined based on comparing each sample point to its eight neighbors in current image and nine neighbors in two adjacent scale images. The gradient magnitude $M\left( {x,y} \right)$ and the orientation $\phi \left( {x,y} \right)$ of the interest point is estimated in terms of pixel differences:

2 $$\left\{ {\begin{array}{*{20}l} {M\left( {x,y} \right) = \sqrt {\left( {L\left( {x + 1,y} \right) - L\left( {x - 1,y} \right)} \right)^{2} + \left( {L\left( {x,y + 1} \right) - L\left( {x,y - 1} \right)} \right)^{2} } } \hfill \\ {\phi \left( {x,y} \right) = \tan^{ - 1} {{\left( {L\left( {x,y + 1} \right) - L\left( {x,y - 1} \right)} \right)} \mathord{\left/ {\vphantom {{\left( {L\left( {x,y + 1} \right) - L\left( {x,y - 1} \right)} \right)} {\left( {L\left( {x + 1,y} \right) - L\left( {x - 1,y} \right)} \right)}}} \right. \kern-0pt} {\left( {L\left( {x + 1,y} \right) - L\left( {x - 1,y} \right)} \right)}}} \hfill \\ \end{array} } \right.$$

where, $L\left( {x,y} \right) = G\left( {x,y} \right) \otimes I\left( {x,y} \right)$. The gradient magnitudes and orientations of the adjacent pixels around the candidate interest point are used to construct the gradient-orientation histogram. In experiments, 4×4 arrays of 8 bin histogram is used, a total of 128-dimensional SIFT descriptor for representing the key point [32].

### Pyramid histogram of orientated gradient (PHOG) descriptor

PHOG is a spatial pyramid extension of the histogram of gradients (HOG) descriptors. HOG is an effective method to characterize the target edge or gradient orientation by extracting the magnitude and orientation of gradient distribution in a localized area of an image $I\left( {x,y} \right)$. Magnitude $M\left( {x,y} \right)$ and orientation $\phi \left( {x,y} \right)$ of the gradient on a pixel are computed as:

3 $$\left\{ {\begin{array}{*{20}l} {M\left( {x,y} \right) = \sqrt {\left( {\frac{{\partial I\left( {x,y} \right)}}{\partial x}} \right)^{2} + \left( {\frac{{\partial I\left( {x,y} \right)}}{\partial y}} \right)^{2} } } \hfill \\ {\phi \left( {x,y} \right) = \tan^{ - 1} \left( {\frac{{\partial I\left( {x,y} \right)}}{\partial x}/\frac{{\partial I\left( {x,y} \right)}}{\partial y}} \right)} \hfill \\ \end{array} } \right.$$

Nevertheless, HOG descriptor does not take into account the division of the image at different spatial scales. The PHOG descriptor is computed by using each edge orientation weighted according to its magnitude at different spatial levels. PHOG descriptor extend HOG descriptor for descriptions of the global shape and the local details of image [4].

### Support vector machine (SVM)

SVM aims to assign labels to instances based on the binary SVM, where the labels are drawn from a finite set of several elements. Given training dataset $\Lambda$, a set of $N$ points is:

$$\Lambda = \left\{{\left.{\left( {x_{i} ,y_{i} } \right)} \right|x_{i} \in R^{p} ,y_{i} \in \left\{ {1,2, \ldots M} \right\}} \right\}_{i = 1}^{N}$$

where $y_{i}$ belongs 1 to $M$, indicating the class to which the point $x_{i}$ attaches. The multi-class method builds binary classifiers which distinguish between one of the labels and the rest (one-versus-all). The $i{\text{th}}$ class is trained with all the training instances of the $i{\text{th}}$ class with positive labels, and all the rest with negative labels. The one-versus-all approach takes the advantage of the decision hyper plane $f_{i} \left( x \right) = \omega_{i}^{T} \varphi \left( x \right) + b_{i}$ to evaluate the class by solving the following optimization problem:

4 $$\begin{array}{*{20}l} {{\text{minimize:}}\, \varOmega \left( {\omega ,\zeta_{j}^{i} } \right) = \frac{1}{2}\left\| {\omega_{i} } \right\|^{2} + C\sum {\zeta_{j}^{i} } } \hfill \\ {{\text{subject to:}}\, 1 - \zeta_{j}^{i} \le \hat{y}\left( {\omega_{i}^{T} \varphi \left( x \right) + b_{i} } \right),0 \le \zeta_{j}^{i} } \hfill \\ \end{array}$$

where $C$ is the tuning parameter and $\zeta_{j}^{i}$ is the slack variable. If $y_{j}$ belongs to the $i{\text{th}}$ class, $\hat{y}_{j} = 1$, otherwise $\hat{y}_{j} = - 1$. Finally, the $i{\text{th}}$ class to which an unknown instance $\hat{x}$ belongs can be determined according the corresponding largest value of $f_{i} \left( x \right)$ [4]:

5 $$\hat{i} = \mathop {\arg \hbox{max} }\limits_{i = 1,2, \ldots ,M} f_{i} \left( x \right) = \mathop {\arg \hbox{max} }\limits_{i = 1,2, \ldots ,M} \omega_{i}^{T} \varphi \left( x \right) + b_{i}$$

### CNNs architecture

**Fig. 1:**
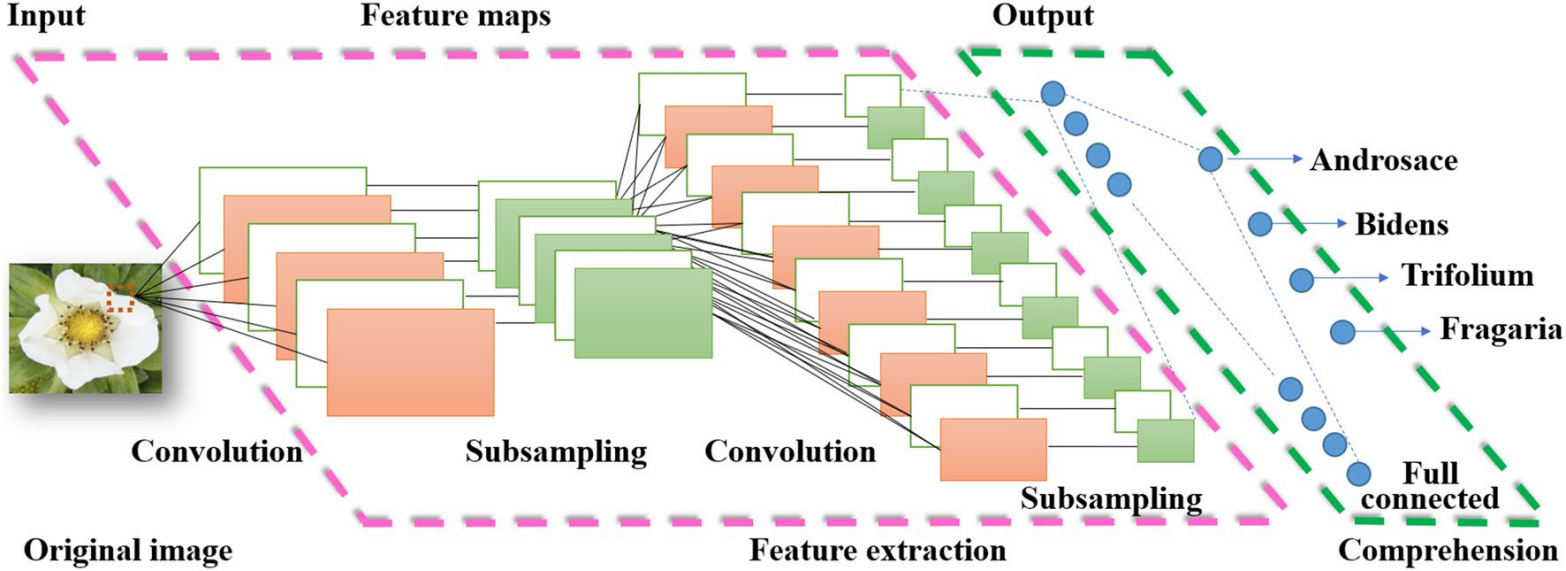
The overall deep architecture of convolutional neural network for detecting four species of white flower species including *Androsace umbellata* (Lour.) Merr., *Bidens pilosa* L., *Trifolium repens* L. and *Fragaria* × *ananassa.* The arrangement of system is presented from left to right in the order: original image data is waiting for analysis at the input level on the left, the feature extraction procedure is performed in the middle layer surrounded the pink dashed rectangle and the determined flower attributes are completed in the final level bounded right green dashed rectangle

The typical CNN for classification target usually consists of the architecture of the input layer, convolutional layers, rectified linear units (ReLU) layers, pooling layers, fully connected layers and dropout layer [10, 35]. The overall deep architecture of CNN for detecting four species of white flowers including *Fragaria* × *ananassa, Androsace umbellata* (Lour.) Merr., *Bidens pilosa* L. and *Trifolium repens* L. are illustrated in Fig. 1. The network specifies the fixed 227 × 227 pixels of a true color image as its input. The following convolutional operation estimates the outcome of neurons connect to local regions in the input layer. The input image is to be convolved with 96 filters of receptive field size 11 × 11 × 3 at stride 4. Iterating this process in the input at stride of 4 gives 55 locations along both width and height, leading to an output matrix of size 11 × 11 × 3 × 55 × 55. The result of a convolution is equivalent to performing one large matrix multiply, which evaluates the dot product between every filter and every receptive field location. The output of this operation would be 96 × 55 × 55, giving the output of the dot product of each filter at each location. The next ReLU layer uses an elementwise maximum value activation function with thresholding at zero. The ReLU is presented to take the place of the earlier standard Sigmoid units in the architecture of neural networks, because the classical Sigmoid function sometimes produces the vanishing gradient issues when calculating the derivative in the saturating region. The ReLU function avoids such issues over and learns much faster than the Sigmoid function, so it was arranged after each and every convolutional and fully-connected layers. The following pooling layer will take a downsampling action along the width and height spatial dimensions. The subsequent fully connected layer is employed to produce a category score corresponding to the input attributes. In this layer each neuron will be linked to all the numbers in the previous neurons. The final dropout layer appears after every fully connected layer. It separately applied a probability score at every neuron of the response map and randomly switches off the activation with the probability to diminish the over-fitting problems. The mentioned deep structure of CNNs for will be applied to automatic discrimination between the *Fragaria* × *ananassa* flowers and other similar white wild flowers in fields.

### Feature extraction

The first step of pipeline of a standard CNN architecture is the feature extraction. CNN deals with an input white flower image and uses a convolutional feature map $\phi^{H \times W \times D}$ with the input image to generate different level features for the final classifiers, where the parameters of $H$, $W$ and $D$ are the height, width and the number of filters. In order to quickly learn effective features in a new classification task using a relative small number of training images, we use the transfer learning methods to fine tune the pre-trained network. This training method is usually much faster and easier than training a network with randomly initialized weights from scratch. Most of these have been trained on the ImageNet dataset, which has 1000 object categories and 1.2 million training images. An analogous illustration has been used previously in discriminative tasks taking on high recognition performance based on CNNs related detectors. Thereby, the network structure originally trained on ImageNet for the task of image classification is used for the feature extraction [20]. The layers property of the CNN architecture is listed Table 2. The network consists of twenty-five layers, which are summarized into 8 layers according to the local function to process the features. There are eight folds with learnable weights comprising of five convolutional layers and three fully connected layers.

**Table 2:** Layers property of the CNN architecture. The network consists of twenty-five layers. There are eight layers with learnable weights: five convolutional layers, and three fully connected layers

No.	Layer name	Description
1	Image input	227 × 227 × 3 true color images with zerocenter standardization
2	1st-level convolution	96 channels, 11 × 11 × 3 convolutions
3	ReLU	Rectified linear units
4	Cross channel standardization	Cross channel standardization with 5 channels per element
5	Max pooling	3 × 3 max pooling
6	2nd-level convolution	256 channels, 5 × 5 × 48 convolutions
7	ReLU	Rectified linear units
8	Cross channel standardization	Cross channel standardization with 5 channels per element
9	Max pooling	3 × 3 max pooling
10	3rd-level convolution	384 channels, 3 × 3 × 256 convolutions
11	ReLU	Rectified linear units
12	4th-level convolution	384 channels, 3 × 3 × 192 convolutions
13	ReLU	Rectified linear units
14	5th-level convolution	256 channels, 3 × 3 × 192 convolutions
15	ReLU	Rectified linear units
16	Max pooling	3 × 3 max pooling
17	6th-level fully connected layer	4096 fully connected layer
18	ReLU	Rectified linear units
19	Dropout	50% of dropout
20	7th-level fully connected layer	4096 fully connected layer
21	ReLU	Rectified linear units
22	Dropout	50% of dropout
23	8th-level fully connected layer	4 fully connected layer
24	Softmax	Softmax
25	Comprehension output	Crossentropyex with *Androsace umbellata* (Lour.) Merr., *Bidens pilosa* L., *Trifolium repens* L. and *Fragaria* × *ananassa*

### Stochastic gradient descent method

The algorithm of gradient descent [28] is performed to optimize the network parameters in order to minimize the back-propagation error on the training dataset. The gradient descent algorithm updates the parameter vector so as to minimize the loss function by taking small steps in the direction of the negative gradient of the loss function:

6 $$\chi_{i + 1} = \chi_{i} - \lambda {\nabla }\psi \left( {\chi_{i} } \right)$$

where $\lambda$ is the learning rate, $\chi$ is the parameter vector, $\psi \left( \chi \right)$ is the loss function and $i$ denotes the iteration number. The standard gradient descent algorithm sometimes oscillates along the steepest decreasing route to search the optimum. In order to reduce the oscillation, a momentum item is supplemented to the above gradient descent function:

7 $$\chi_{i + 1} = \chi_{i} - \lambda {\nabla }\psi \left( {\chi_{i} } \right){ + }\tau \left( {\chi_{i} { - }\chi_{i - 1} } \right)$$

where $\tau \in \left[ {0,1} \right]$ is the momentum coefficient. The normal gradient descent algorithm estimates the gradient of the loss function $\psi \left( \chi \right)$ using the entire dataset at once. The stochastic gradient descent algorithm estimates the gradient of the loss function $\psi \left( \chi \right)$ and renews the parameters using a stochastic subset of the dataset. In this paper, the number of stochastic subset using to train the CNN model is set as 10.

### Training networks

**Fig. 2:**
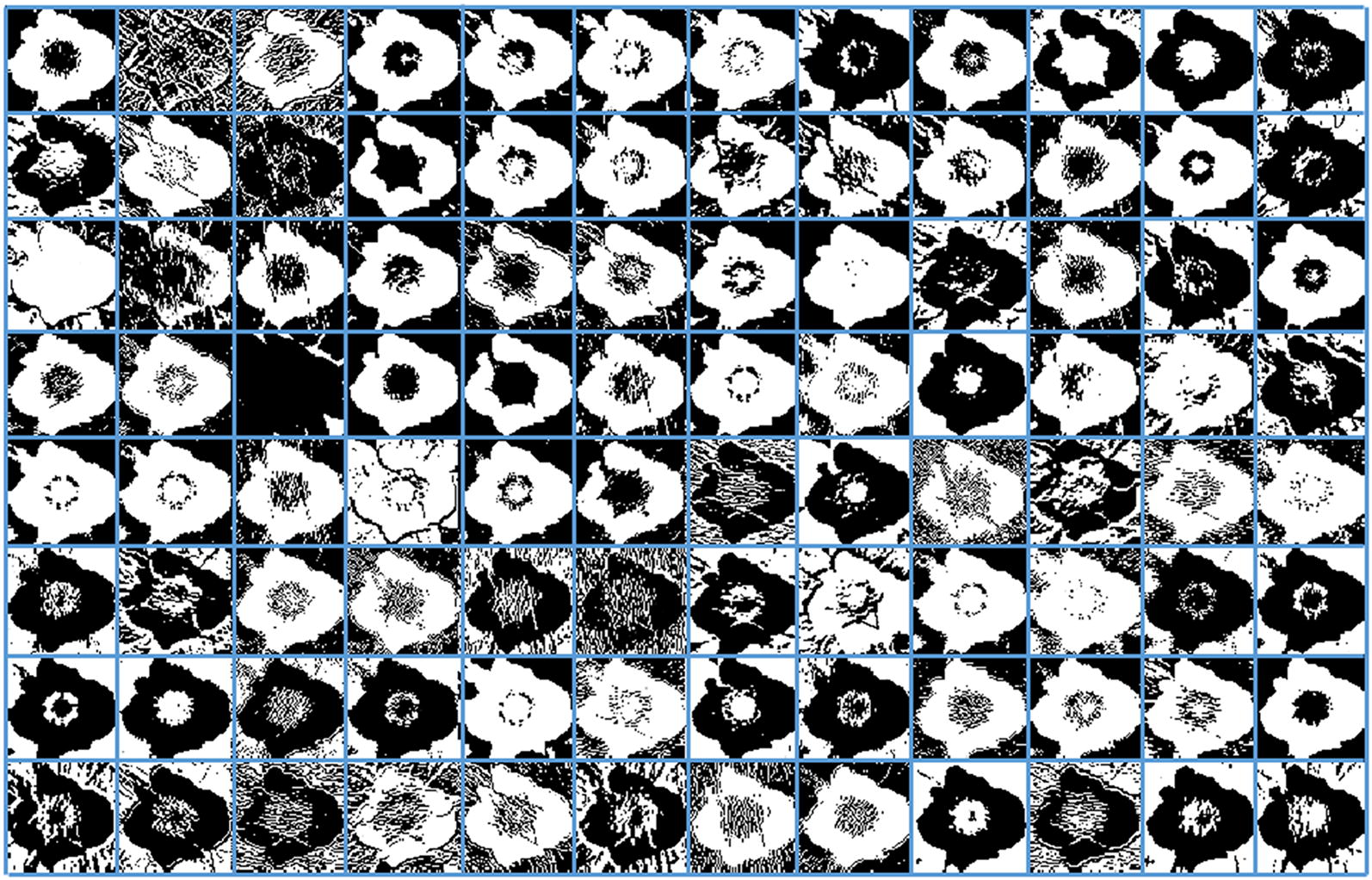
Illustrate the 96 channels of captured rich structure and texture feature information from the *Fragaria* × *ananassa* flower image in the first convolutional layer by using size of 11 × 11 convolutional kernels. These images contain from a different variety of frequency-, orientation- and color-selective features

The CNN uses a receptive field-like layout in which each neuron receives connections only from a subset of neurons in the lower layer. The receptive field of a neuron in one of the lower layers encompasses only a small region of the image. The convolutional layer is sensitive to the size of receptive field of image. When the original image sizes are around 200 × 200–700 × 700, the area of receptive field can be set between the sizes of 7 × 7 and 15 × 15 [27]. The neurons of structure properties are sometimes generated by using the large convolutional kernels, while the texture properties are captured by using small convolutional kernels. Generally, the decent size kernels might reach the balance between two tendencies. Figure 2 illustrates the 96 channels of captured rich structure and texture feature information from the *Fragaria* × *ananassa* flower image in the first convolutional layer by using size of 11 × 11 convolutional kernels. These images contain from a different variety of frequency-, orientation- and color-selective features. There were 256, 384, 384 and 256 channels of captured more rich structure and texture feature information from the second to fifth convolutional layer. The layers in the network can produce more complex structure and texture features of flower image for the subsequent neurons. These features further exhibit the superior performance in the task of identifying the white flower images.

## Results and discussion

### Momentum parameter determination

**Fig. 3:**
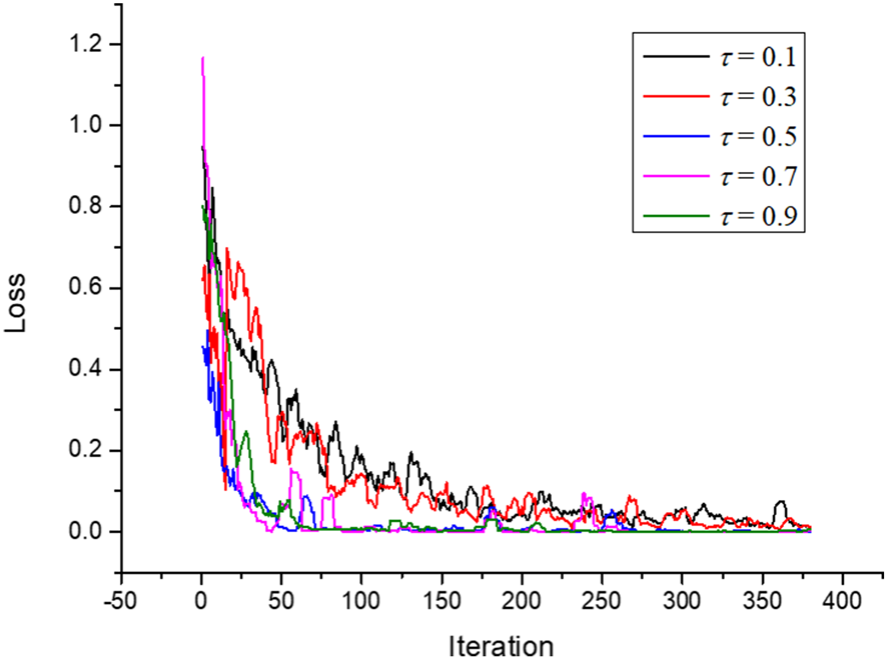
Five curves of training loss function of a twenty-five-layer architecture of convolutional neural network in the iteration optimization process with momentum coefficients of $\tau$ = 0.1, 0.3, 0.5, 0.7 and 0.9 on the white flower dataset

Figure 3 shows 5 curves of training loss function $\psi \left( \chi \right)$ of a twenty-five-layer architecture of CNN in the iteration optimization process with momentum coefficients of $\tau$ = 0.1, 0.3, 0.5, 0.7 and 0.9 on the white flower dataset. The correct use of stronger momentum (as determined by $\tau$) had a dramatic effect on optimization performance for the CNNs. The momentum item is actually the contribution of the previous gradient change. It can be seen that the contribution of the gradient changes from the previous iteration to the current iteration in the training set greatly affects the convergence of the loss function. Along with the growth of momentum coefficient values from $\tau$ = 0.1, 0.3 and 0.5 the convergence performance is gradually improved. Along with the growth of momentum coefficient values from $\tau$ = 0.7 and 0.9 the convergence performance become worse. It indicates that the attached momentum item is able to reduce the oscillation when algorithm searches the optimum along the convex route. Although the convergence speed of curve with $\tau$ = 0.7 is faster than the one with $\tau$ = 0.5 at the beginning stage, the convergence performance of curve with $\eta$ = 0.7 obviously shocks severely at the iteration locations between 50 and 90. Thereby, the momentum parameter $\tau$ = 0.5 in the stochastic gradient descent function is chosen for training the CNN model.

### Accuracy by CNNs

**Fig. 4:**
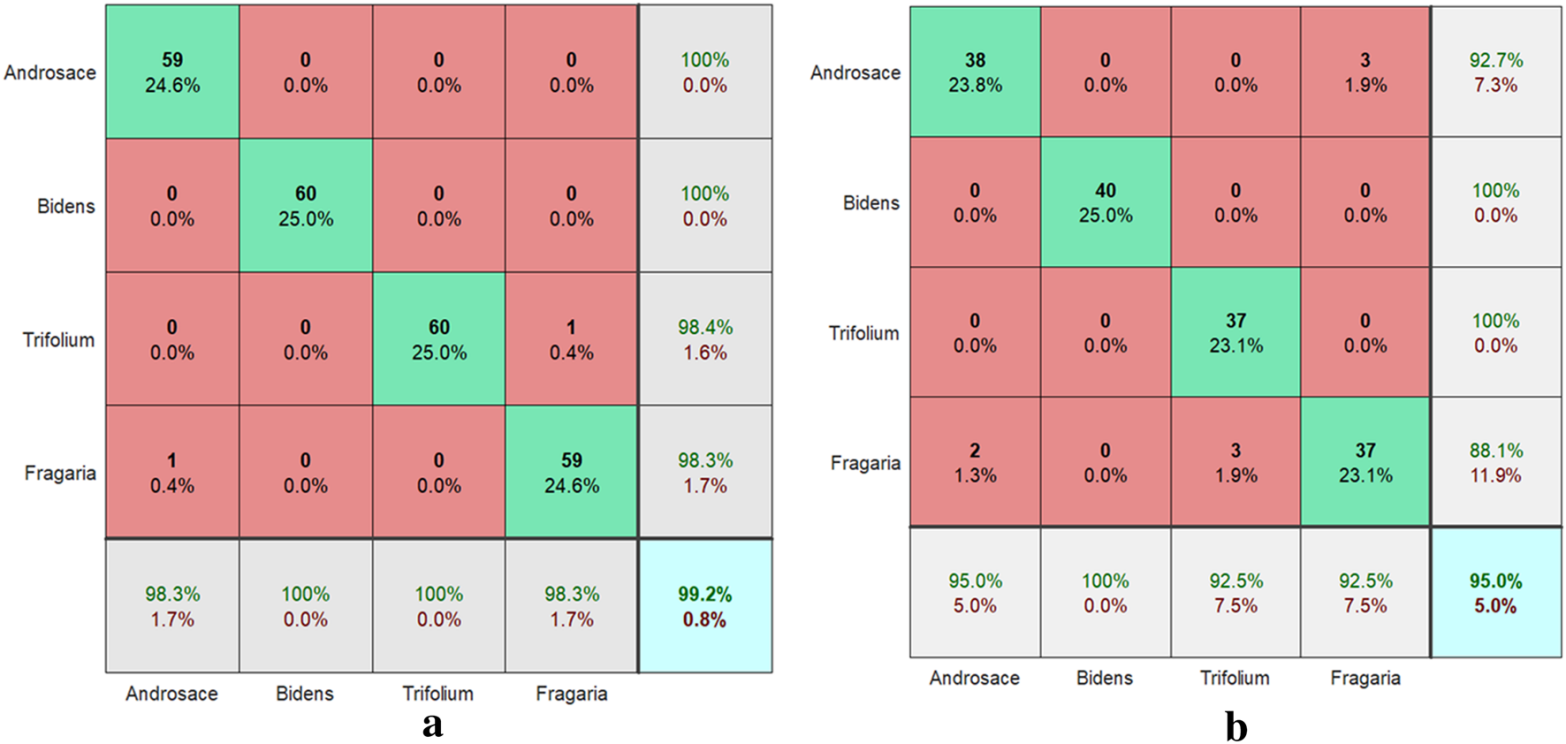
Confusion matrix diagrams of discriminating four different species of white flowers of *Androsace umbellata* (Lour.) Merr., *Bidens pilosa* L., *Trifolium repens* L. and *Fragaria* × *ananassa images* based on deep learning artifices of convolutional neural network on the training (**a**) and test (**b**) dataset, respectively

The bottom layer of the CNN framework was used as filters for capturing blob and edge features. These primary features were then processed by deeper network framework, which combined the early features to form higher-level semantic features. These higher-level semantic features were better suited for following recognition tasks [7]. In this paper, we used a multiclass SVM classifier at the top of the CNN-based classification architecture for training the high-level CNN image features. The stochastic gradient descent algorithm was used for speeding up the training the high-dimensional CNN feature vectors. Firstly, we presented the accuracy achieved by using such CNN architecture. The training CNN work was implemented offline, i.e., before employing CNN for the classification of 240 white flower images. The identification process itself performed species identification on 160 white flower images. The confusion matrix [9] diagram is employed to summarize and visualize the results of the performance of an algorithm of classification performance of white flower using the CNN algorithm. As shown on Fig. 4, the rows indicate the output class (predicted class), and the columns correspond to the target class (actual class). The green diagonal elements show for the number and the corresponding percentage of the instances where the CNN models correctly measure the categories of white flowers. For the training set, 59, 60, 59 and 59 objects are correctly identified as the flower classes of *Androsace umbellata* (Lour.) Merr., *Bidens pilosa* L., *Trifolium repens* L. and *Fragaria* × *ananassa*, respectively. These corresponds to 24.6, 25.0, 25.0 and 24.6% of all 240 training white flower images, respectively. Similarly, for the test set, 38, 40, 37 and 37 objects are correctly classified as the flower classes of *Androsace umbellata* (Lour.) Merr., *Bidens pilosa* L., *Trifolium repens* L. and *Fragaria* × *ananassa*, respectively. These corresponds to 23.8, 25.0, 23.1 and 23.1% of all 160 test white flower images, respectively. The red non-diagonal elements show where the model has made wrong prediction. For the training set, out of 60 *Androsace umbellata* (Lour.) Merr. cases, 1 object is mistakenly discriminated as the species of *Fragaria* × *ananassa*. This corresponds to 0.4% of all 60 *Androsace umbellata* (Lour.) Merr. instances. Out of 60 *Fragaria* × *ananassa* cases, 1 object is mistakenly detected as *Trifolium repens* L. instance. This corresponds to 0.4% of all 60 *Trifolium repens* L. objects. All of 60 *Bidens pilosa* L. objects and 60 *Trifolium repens* L. objects are correctly identified. Similarly, for the test set, out of 40 *Androsace umbellata* (Lour.) Merr. cases, 2 objects are mistakenly discriminated as the species of *Fragaria* × *ananassa*. These correspond to 1.3% of all 40 *Androsace umbellata* (Lour.) Merr. instances. Out of 40 *Trifolium repens* L. cases, 3 objects are mistakenly detected as *Fragaria* × *ananassa* instances. These correspond to 1.9% of all 40 *Fragaria* × *ananassa* objects. Out of 40 *Fragaria* × *ananassa* cases, 3 objects are mistakenly detected as *Androsace umbellata* (Lour.) Merr. instances. These correspond to 1.9% of all 40 *Fragaria* × *ananassa* objects. All of 40 instances of *Bidens pilosa* L. objects are correctly identified. The column with the white background on the far right of the diagram shows the accuracy for each output class. For the training set, all of 59 *Androsace umbellata* (Lour.) Merr. and 60 *Bidens pilosa* L. predictions, 100% are true. Out of 61 *Trifolium repens* L. predictions, 98.4% are true and 1.6% are false. Out of 60 *Fragaria* × *ananassa* predictions, 98.3% are true and 1.7% are false. Similarly, for the test set, all of 40 *Bidens pilosa* L. and 37 *Trifolium repens* L. predictions are true. Out of 41 *Androsace umbellata* (Lour.) Merr. predictions, 92.7% are true and 7.3% are false. Out of 42 *Fragaria* × *ananassa* predictions, 88.1% are true and 11.9% are false. The row with the white background at the bottom of the diagram shows the accuracy for each target class. Out of 60 *Androsace umbellata* (Lour.) Merr. and *Fragaria* × *ananassa* cases, 98.3% are correctly predicted as themselves and 1.7% are predicted as the false instances, respectively. All of 60 instances of *Bidens pilosa* L. and 60 *Trifolium repens* L. objects are correctly identified as themselves. Similarly, for the test set, out of 40 *Androsace umbellata* (Lour.) Merr., 40 *Trifolium repens* L. and 40 *Fragaria* × *ananassa* cases, 95.0, 92.5 and 92.5% are correctly predicted as themselves and 5.0, 7.5 and 7.5% are predicted as the false instances, respectively. All of 40 instances of *Bidens pilosa* L. objects are correctly identified as themselves. The bright blue elements in the bottom right of the diagram illustrate the overall accuracy of the algorithm. Overall, 99.2 and 95.0% of the predictions are true and 0.8 and 5.0% are false on the white flowers training and test set, respectively.

### Comparing performance of algorithms

**Fig. 5:**
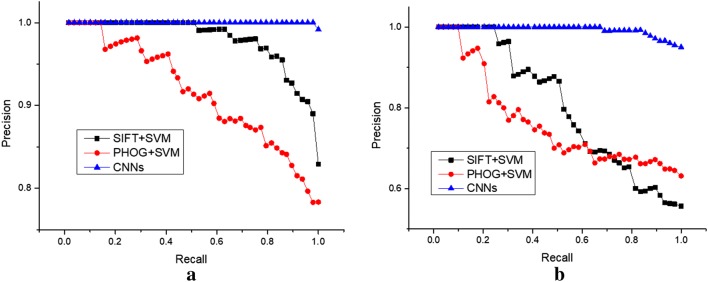
Precision-recall curves of detecting four species of white flowers including *Androsace umbellata* (Lour.) Merr., *Bidens pilosa* L., *Trifolium repens* L. and *Fragaria* × *ananassa* based on deep learning methods of convolutional neural networks on the training (**a**) and test (**b**) dataset, respectively

The precision-recall metric [29] is used to estimate the algorithm quality of detecting the flower varieties. The precision-recall curve shows the tradeoff between precision and recall for different threshold. The high precision relates to a low false positive rate, and high recall relates to a low false negative rate. The large scores indicate that the classification model is returning accurate results as well as returning a majority of all positive results. We compared our method with category discovery methods of SVM combined with the traditional hand-engineered features of SIFT and PHOG. As shown in Fig. 5, as the threshold of recall rates increase, the corresponding precision rates of CNN are much higher than other two algorithms of SIFT + SVM and PHOG + SVM. The overall performance of the algorithms is measured with the mean average precision (mAP) score [12], which is the average precision at the ranks where recall changes. The geometric interpretation of mAP score is the area below the curve. A large area under the precision-recall curve denotes the overall superior performance of algorithm with the high mAP score. The CNN-based model achieves the highest mAP scores of 0.983 and 0.974 on the training and test flower image dataset, respectively (See Table 3). The compared results illustrated that the improvement of the proposed model for classification of the white flower images with complex background on both of the training and test dataset is substantial. It appears that, more detailed features are abstracted effectively from the original images of white flowers by using the deep learning methods of CNNs compared with other two algorithms.

**Table 3:** Accuracy and mean average precision (mAP) scores of detecting four species of white flowers including*Androsace umbellata* (Lour.) Merr., *Bidens pilosa* L., and *Trifolium repens* L. and *Fragaria* × *ananassa* based on deep learning methods of convolutional neural network on the training and test dataset, respectively

Method	Training set		Test set	
	Accuracy (%)	mAP	Accuracy (%)	mAP
SIFT + SVM	82.9	0.960	55.6	0.794
PHOG + SVM	78.3	0.900	63.1	0.744
CNNs	99.2	0.983	95.0	0.974

In order for flower recognition task to be implemented, the algorithm must have good ability in dealing with variability of flower appearance. The SIFT feature descriptor is invariant to uniform scaling, orientation and illumination changes. The SIFT descriptors are estimated at points on a regular grid over the foreground flower patch. At each grid point the descriptors are computed over circular support patches. Key points are defined as maxima and minima of the result of difference of Gaussians function applied in scale space to a series of smoothed and resampled images. A 128-dimensional feature vector are generated from the grey image to indicate the flower. The histogram of gradients descriptor technique counts occurrences of gradient orientation in localized portions of an image. The PHOG descriptors are spatial pyramid extension of the histogram of gradients descriptors. Thus, the local object appearance and shape of a flower image can be described by the distribution of intensity gradients or edge directions. The SIFT and PHOG features are further used as the input feature vectors of the nonlinear learning machine of multi-class SVM. The classification results of three kinds of methods are listed in Table 3. The algorithm of SIFT + SVM attains the comprehending accuracy in the training and test sets are 82.9 and 55.6%, respectively. The algorithm of PHOG + SVM achieves the detection accuracy in the training and test sets are 78.3 and 63.1%, respectively. The identification accuracy of CNNs is 99.2 and 95.0% in the training and test procedure, respectively, which is much higher than the above two methods. The SIFT and HOG features are low-level features which don’t make use of hierarchical layer-wise representation learning while the CNN is a hierarchical deep learning model which is able to learn low-level features similar to SIFT and HOG features from training examples alone for more and more abstract representations. The multi-level deep convolutional structure can attain more detailed features from images and improving the accuracy of measurement results. The state-of-the-art proposal methods provides a superior alternative for the precise classification of the white flowers *of Fragaria* × *ananassa* from other three wild species of *Androsace umbellata* (Lour.) Merr., *Bidens pilosa* L. and *Trifolium repens* L. in fields.

## Conclusions

In this investigation, we have presented a CNN architecture for the deeply classifying four species of white flowers including *Androsace umbellata* (Lour.) Merr., *Bidens pilosa* L., *Trifolium repen* L. and *Fragaria* × *ananassa*. The CNN-based algorithm achieved outstanding 99.2% training and 95.0% test accuracy in the application of identifying the white flower images, respectively. The proposed model in this study turns out to be much more accurate than traditional models of SIFT + SVM and PHOG + SVM. The state-of-the-art proposal CNN method demonstrated an artificial intelligence capable of precise classification of the white flower images with a level of competence comparable to general algorithms. Our team plans to enlarge current flower dataset which will consist of more wild flower species and numbers. Further research is also necessary to evaluate performance in a real-time detection setting, in order to validate this technique across the full distribution and spectrum of *Fragaria* × *ananassa* flower fields encountered in typical practice. The technologies can be potentially used to quickly and exactly check the number of strawberry flowers in fields from the images captured from unmanned ground vehicle.
